# Integrating design thinking into dental education

**DOI:** 10.3389/froh.2025.1547335

**Published:** 2025-03-13

**Authors:** Supachai Chuenjitwongsa, Lisa R. Amir, Abbas Jessani, Lakshman P. Samaranayake, Thanaphum Osathanon

**Affiliations:** ^1^ Dental Education Unit, Office of Academic Affairs, Faculty of Dentistry, Chulalongkorn University, Bangkok, Thailand; ^2^ Dental Education Unit and Department of Oral Biology, Faculty of Dentistry, Universitas Indonesia, Jakarta, Indonesia; ^3^ Division of Dental Public Health, Schulich School of Medicine and Dentistry, Western University, London, ON, Canada; ^4^ Department of Epidemiology and Biostatistics, Schulich School of Medicine and Dentistry, Western University, London, ON, Canada; ^5^ Office of Research Affairs, Faculty of Dentistry, Chulalongkorn University, Bangkok, Thailand; ^6^ Faculty of Dentistry, The University of Hong Kong, Hong Kong, Hong Kong SAR, China; ^7^ Center of Excellence for Dental Stem Cell Biology, Department of Anatomy, Faculty of Dentistry, Chulalongkorn University, Bangkok, Thailand

**Keywords:** dental education, design thinking, curriculum, faculty development, teaching, learning

## Abstract

Design thinking is a human-centred, iterative process that aims to develop innovative solutions tailored to user needs. This article examines the groundwork and incorporation of design thinking in healthcare and medical education, highlighting its potential benefits in dental education, including enhancements in learner-centred approaches, faculty development, interprofessional collaboration, and person-centred care. Design thinking methods foster learner engagement, aligning with cognitive and constructivist learning theories. Active engagement and discourse among learners create meaningful learning experiences, benefiting from a “learning by doing” approach. Further, design thinking processes ensure critical thinking and collaborative learning, supporting active engagement with prior knowledge and constructive feedback skills. Thus, applying design thinking in dental education could deepen learners' understanding with improved problem-solving skills, ultimately leading to effective learning outcomes.

## Introduction

Design thinking is a process used in numerous applications to foster practical solutions to develop or innovate for users' specific needs (1). It is a structured and repetitive approach that initiates with a deep understanding of user needs, which is stated as an emphatizing step. Then, the process includes defining a problem, ideating an innovative solution, creating a prototype, and testing and evaluating it. This approach is supported by cognitivist and constructivist theories (2, 3). The design thinking concept is commonly utilised in business expansion activities, such as product development. Additionally, it is also applied in other fields, such as in health care and medical education (4, 5).

In medical education, design thinking has been applied in the creation of several crucial components, such as patient management (6), curriculum development (5), learning experience design (4), and faculty development (7). Nonetheless, research on the implementation of design thinking, particularly in dental education, remains limited (8). Hence, this article focuses on examining the viability of incorporating design thinking principles within the realm of dental education.

## The concept of design thinking

**Figure 1:**
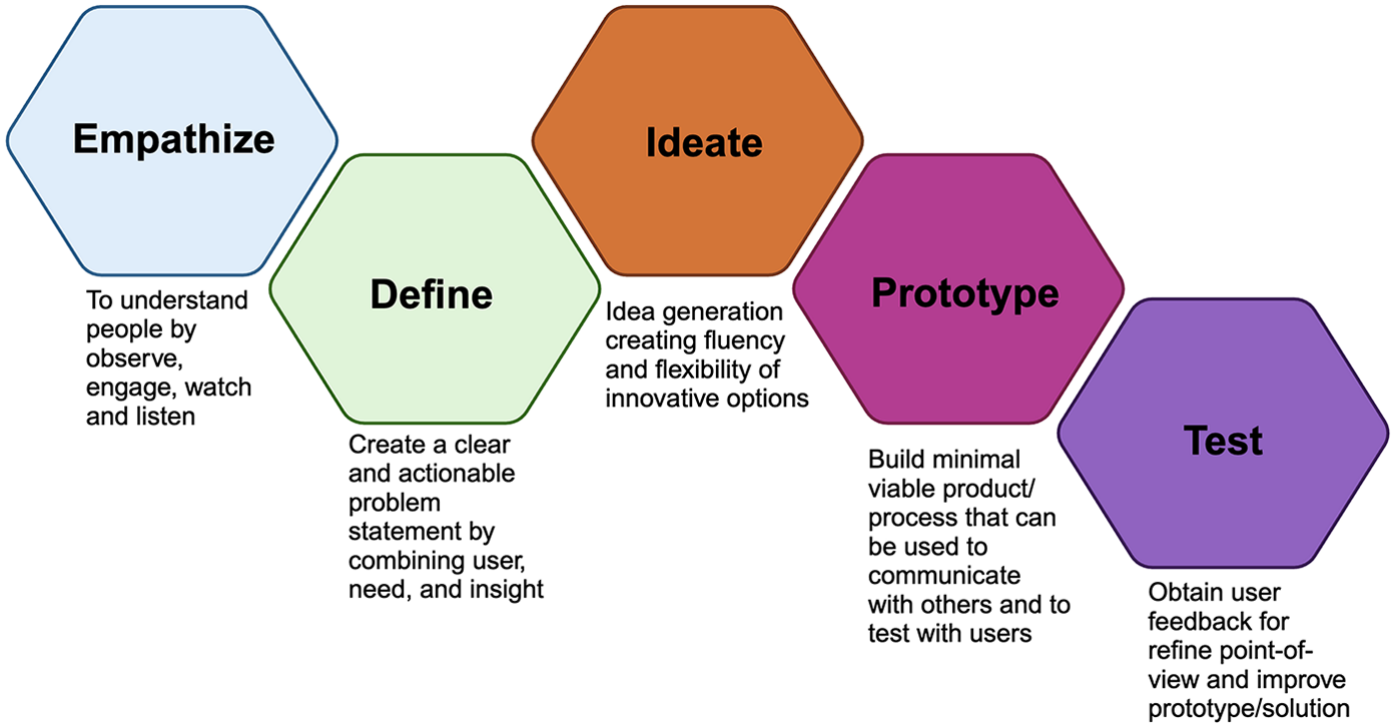
Schematic diagram demonstrates five steps of design thinking (9). Created by Biorender.com.

The concept of design thinking was enunciated in 2008 by Tim Brown in a paper published in the *Harvard Business Review,* wherein he described three stages of design thinking: inspiration, ideation, and implementation (1). First, the inspiration stage aims to consolidate a problem and/or opportunity. Next, the ideation stage focuses on creating and developing various ideas tailored to find the solutions for the identified problem, and finally, the implementation stage aims to apply the solution. These could be amplified into the following five steps: (i) empathise, (ii) define, (iii) ideate, (iv) prototype, and (v) test, which are systematically planned and iteratively utilised (Figure 1) (9), This prevailing design thinking model traces its roots to the Hasso Plattner Institute of Design, Stanford School in the USA.

## The five steps of design thinking

The five steps of design thinking are further clarified below.

1. **Empathise Phase.** This user-centric approach places a significant emphasis on the empathy step, aiming to profoundly engage with users' experiences to gain a thorough understanding of their needs (10). Several methods are introduced as tools for empathising. Observation stands out as a simple yet potent method. Direct observation provides insights into people's behaviors and aids in predicting their intentions. Engaging in an in-depth conversation with the participants can also provide meaningful insights. This phase allows the observer to transcend his/her assumptions and biases and unwaveringly focus on the genuine needs of the users. For instance, some healthcare companies have used an extensive empathising process to understand the need to re-design the nursing-staff shift change process, eventually leading to an innovative process that improves patients' experience and nurse satisfaction and productivity (1). Dental education primarily revolves around chairside teaching, where students must perform complex, often irreversible procedures while effectively communicating with patients from diverse backgrounds and with varying needs (11). Consequently, dental students face the challenge of balancing technical excellence with meeting patient expectations. Design thinking in dental education can be applied in conjunction with chairside teaching. During the empathise phase, educators observe students' needs and interactions within various dental educational settings, including laboratory and clinical environments. This observation phase could reveal students' stress levels and overwhelming experiences throughout their study period. The gathered insights serve as crucial input for subsequent phases of design thinking, enabling the development of an innovative dental curriculum that addresses students' pain points and enhances their psychological well-being during their dental education journey. Another example of the use of the empathise phase in dental education development is educators performing systematic observation in clinical sessions. Direct shadowing provides observational information, for example, on students struggling with clinical procedures, handling clinical equipment, adjusting patient positioning, time management, or experiencing anxiety during clinical treatment sessions. This could be beneficial fundamental feedback for developing targeted and innovative clinical teaching methods.
2. **Define phase.** the second step in the design thinking process involves synthesizing and organizing data to uncover connections and patterns, which can then be used to articulate a clear and concise problem statement. The define step can also yield a point of view (POV) statement, which is a specific user problem that needs to be addressed. POV must be discreet and focused, well framed and articulated to inspire the team, establish criteria for generating ideas, and empower the team to make decisions independently (9). A well-defined problem statement is essentially fundamental for the subsequent ideation process so as to target the identified problems/needs precisely. After shadowing dental students in clinical sessions during the empathise phase, educators may identify various aspects that require innovative approaches. To address these challenges effectively, a clear point of view (POV) must be formulated to precisely determine and define the problem. For instance, observations may reveal that dental students need strategies to manage their anxiety during clinical practice sessions, as it can hinder their performance and competency development. Formulating a clear POV is beneficial in later stages of the design thinking process, as it helps educators focus on developing targeted, innovative solutions to address the identified issue.
3. **Ideate phase.** A well-defined problem statement advances to this particular stage where all possible solutions are explored. It is crucial in this stage that the idea must be generated according to the gathered information without any preconceived judgments or biases (9, 10). Various combinations of techniques can be used in the ideation process, including brainstorming, prototyping, body storming, mind mapping, and sketching (9). To address the aforementioned POV regarding student anxiety in clinical practice sessions, educators and students can collaborate in brainstorming sessions to generate ideas. During these ideation meetings, it is crucial to foster a non-judgmental and open-minded atmosphere, encouraging participants to propose a wide range of diverse ideas without initially considering their feasibility or limitations. Some potential ideas for improving student anxiety in clinical practice sessions include developing short role-playing scenarios for students to practice patient interactions prior to clinical sessions, pairing students to support each other during clinical sessions, utilizing virtual reality technology to simulate patient interactions and help students practice in a controlled environment, and designing clinical cases with progressive difficulty levels to help students build confidence gradually, and so on. As the number of ideas grows, educators can then proceed to the next phase of the design thinking approach to further refine and develop the most promising solutions.
4. **Prototype phase.** Once all ideas are gathered, critical thinking is applied to discern the most pertinent concepts based on practical considerations such as budget, time constraints, and others. The most relevant idea is then developed into a prototype, which is a tangible and low-fidelity model. This prototype serves as proof of concept to explore and test the application of the idea to address the defined needs. It can be used as a tool for ideating and problem-solving, communicating with teams and users, testing possibilities of the solution, and managing the solution-building process leading to the test phase (9). In the prototype phase, one potential solution to address the POV regarding the reduction of student anxiety in clinical practice sessions mentioned above could be the development of structured clinical cases with progressive difficulty levels. This approach aims to help students build confidence gradually. To create this prototype, educators should identify and describe the case difficulty levels for each discipline, tailoring them to novice, trained, and competent students. For example, novice students may start with simple cases that focus on basic skills and patient interaction, while trained students progress to more complex cases that require advanced techniques and decision-making. Competent students can then tackle the most challenging cases. Once these structured case difficulty descriptions are established, they can be further refined and tested in the subsequent test phase of the design thinking process.
5. **Test phase.** Finally, the prototype is tested to create the user experience and obtain user opinions. This feedback helps to identify the advantages and disadvantages of the prototype and is further utilised to improve it. Feedback provides more engagement and information from users and refines POV (9). This repetitive cycle refines and optimises the solution to get the best fit of the solution to users' needs (1). The exemplification of the test phase for POV improving student anxiety in clinical practice is as follows. The structured clinical cases prototype is implemented and evaluated in the test phase to assess its effectiveness in reducing student anxiety. During the employment of this approach, educators monitor their performance and anxiety levels and collect feedback from students and faculty. The data gathered should be analysed to identify areas of success and opportunities for improvement, leading to refinements in the prototype and further ensuring continuous improvement and validation of the solution's effectiveness.

## Educational foundation of design thinking

Design thinking is underpinned by cognitivist and constructivist theories, which provide a robust framework for understanding the learning processes involved in this approach (2, 3). Cognitivism suggests that learning is intricately linked to higher-order cognitive processes, such as critical, logical, and analytical thinking (12). These cognitive processes are fundamental to each phase of design thinking, enabling learners to critically formulate ideas and problem statements, analyze user needs, and evaluate the results of prototype testing. Through active engagement in retrieving prior knowledge, learners are able to contextualize the identified challenges, leading to intelligent and efficient problem-solving. This process of linking new information to existing knowledge fosters a deeper understanding, leading to profound learning outcomes (13).

Constructivism further emphasizes that learning is constructed through individual interpretation, meaning-making, and experiential engagement (13). Within the design thinking framework, learners have opportunities to expand their existing knowledge while acquiring new insights and interpretations. They develop personalized perspectives on proposed or hypothetical problems, actively engage in group discussions with diverse viewpoints and experiences, and explore the synergy between multiple concepts and situations during prototype testing. This higher cognitive and collaborative environment facilitates the generation and integration of new knowledge pertinent to the prototype being tested (3). Therefore, it is imperative for educators to foster active engagement and discourse among learners, thereby creating meaningful learning experiences. Additionally, providing adequate support and feedback is essential to ensure that learners benefit from a “learning by doing” approach (13). Furthermore, educators should ensure critical thinking and collaborative learning, support active engagement with prior knowledge, and provide constructive feedback. This approach will deepen learners' understanding with improved problem-solving skills, ultimately leading to effective learning outcomes.

## Application of design thinking in dental and healthcare education

**Figure 2:**
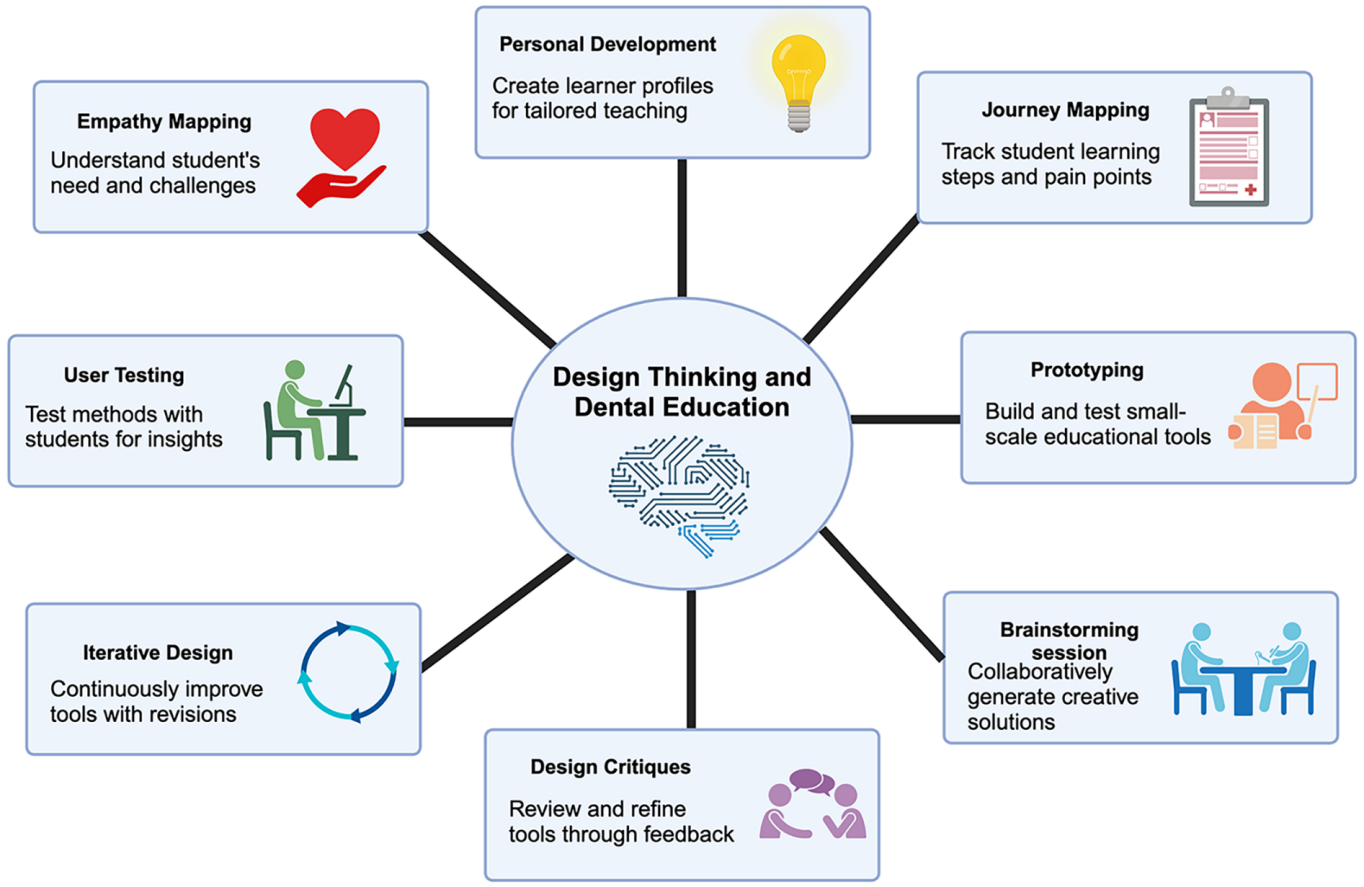
Application of design thinking in dental and healthcare education. (created by biorender.com).

In the field of dental education, design thinking has been advocated to encourage the development of creative-higher-order cognitive processes through innovative teaching pedagogies. These processes could be further enhanced through specific design thinking methods that can be effectively integrated into dental education, including (Figure 2).

(1) *Empathy Mapping:* Encouraging students to empathise with patients by creating empathy maps which help them understand patients' needs, emotions, and experiences better (1). The empathy map has been developed in various contexts and utilised in healthcare education. The Jefferson Scale of Empathy and its modifications, which use a seven-point Likert scale, have been employed in numerous studies. This scale comprises three domains: perspective taking, compassionate care, and standing in the patient's shoes (1–3). Reports have shown that empathy maps can guide nursing students in engaging in empathetic communication with patients, highlighting the value of empathy maps for empathy development (4). In the context of design thinking in dental education, the empathy map can be integrated into the empathise phases. Dental students engage in conversations with patients, asking open-ended questions and observing non-verbal cues to better understand their patients' experiences, thoughts, and emotions. The empathy map can be used for students to gain more insightful information from these conversations, capturing the patient's thoughts, feelings, actions, and emotions in a structured manner. By incorporating empathy mapping into the design thinking process, dental educators can promote patient-centered care, enhance students' communication skills, and foster empathy. This approach encourages dental students to consider their patients' perspectives, leading to improved patient care and satisfaction.
(2) *Persona Development:* Creating detailed patient personas based on different demographics and needs can help students design tailored solutions for specific patient groups (14).
(3) *Journey Mapping:* Mapping out the patient journey from scheduling an appointment to post-treatment follow-up can help identify pain points and areas for improvement in the patient experience (15).
(4) *Prototyping*: Encouraging students to create prototypes of new dental tools, technologies, or processes can help them test and refine their ideas before implementation.
(5) *Brainstorming Sessions:* Conducting brainstorming sessions to generate a wide range of ideas for addressing specific dental care challenges can foster creativity and innovation (16).
(6) *Design Critiques*: Encouraging students to provide constructive feedback on each other's design solutions can promote a culture of collaboration and continuous improvement.
(7) *Iterative Design:* Emphasizing an iterative design process where students refine their solutions based on feedback and testing can help them develop more effective and patient-centred approaches.
(8) *User Testing*: Involving actual patients in the testing and evaluating new procedures/approaches, ranging from oral health promotion to treatment schemes or technologies, can provide valuable insights for improving patient outcomes.

By incorporating these design thinking methods into dental education curricula, educators can help students develop critical thinking, problem-solving, and empathy skills essential for providing high-quality, patient-centered care. The foregoing tools in the student armamentarium are considered valuable in addressing the challenges in dentistry and other health education fields. These encourage learners to work together on critically assessing patient-related problems with an open mind, leading to person-centred innovative solutions. This process encourages meaningful participation and promotes the highest level of learners' and preceptors' engagement (17).

Particularly in dental education, empathising techniques can be useful to develop a deeper understanding of patient experiences, needs, and expectations. Simultaneously, this process fosters the development of higher-order cognitive abilities, enhances the understanding of contextualised situations, and promotes deep learning (2, 3). These immersive experiences not only contribute towards the learner's awareness of oral health but also prepare them to develop appropriate management plans that serve individualised patients' needs.

On example where such design thinking methodology has been illustrated by Delvin et al. (18). They describe clearly how design thinking was utilised to create a new clinical care model that addressed the critical needs of patients during the recent COVID-19 pandemic, for instance, by inclusion of telemedicine/teledentistry program which leads to optimum patient outcomes.

 Design thinking can also be a beneficial tool for identifying the learners' specific educational needs and developing prototypes for their learning experiences. An exemplification of this is the approach in medical education to use design thinking to identify and design the training tools to support virtual ambulatory care learning (19). Similar applications can be adapted in dental curricula with key stakeholders, including learners and educators, who can be engaged in the process of curriculum development.

Design thinking models have been used to design and evaluate dental and oral health education media through web-based platforms. Jumlongkul et al. describe a model where the user is involved in the design process to solve problems from the very early stages so that it encourages the emergence of new ideas form the early design stages until the final testing is carried out. The particular design thinking method applied was a user interface and experience (UI/UX) design to ensure solutions that met user needs (20).

Another study reported the use of an adaptive design thinking model during the COVID-19 pandemic to develop innovation and entrepreneurship courses for dental learners. Research skills and entrepreneurial knowledge are crucial, and their integration into the dental curriculum is essential. These strengthen learners' ability and mindset to face real-world clinical challenges. It is believed that such design thinking activities provide more instructive and durable solutions for healthcare, including dentistry in comparison to traditional problem-solving techniques (21).

 Design thinking can also be used to create faculty development programs. Wolcott provides a good example where design thinking was successfully used to frame the AACP (American Association of Colleges of Pharmacy) Teacher's Seminar review (22) where over 90 content ideas and 50 format options were ideated by the study participants. Of these, several prototypes were selected and tested to guide the final selection for the seminar, where a majority either agreed or strongly agreed that design thinking was a helpful approach to creating the reviewed framework. Similar design thinking programs could be adapted for faculty development programs in dentistry to replace or enrich the traditional lecture-based approaches or workshops. Such novel and refreshing approaches can also serve the evolving needs of the continuing professional development of faculty members.

Finally, in this context, design thinking is an excellent mechanism for fostering interdisciplinary collaboration. This notion is supported by constructivist theory, which emphasizes that learning and knowledge creation occur through collaborative interactions among group members. Practicing active listening without preconceived biases is the first step in training and preparing dental learners to work efficiently in multidisciplinary teams in the real-world healthcare system. This can be accomplished by implementing cross-discipline workshops for design thinking in dentistry and other disciplines into existing roasters instead of adding an additional workload (23).

The evidence for using design thinking in the context of dental specialties is limited due to the lack of studies and research in this area. However, some perspectives can be described on the potential integration of design thinking in education for dental specialties. In pediatric dentistry, for example, students can use the empathise phase to determine patients' needs and identify the hidden reasons behind individual children's behaviors. This can help define patients' problems and be further used to implement innovative approaches to improve patient experience throughout the clinical session. Beyond patient care, design thinking can also be used to enhance dental specialty training. Educators can shadow students during operative procedures and empathise with their experiences. Problems can be identified, such as difficulties in adjusting patient positioning during clinical treatment. This could lead to the ideation of improved concepts for ergonomic teaching, which can be implemented to enhance students' learning experience in clinical settings.

Empathising with students in prosthodontic clinics may reveal struggles with complex crown preparations. This POV can be addressed by numerous ideation approaches, such as the development of augmented reality (AR) or virtual reality (VR) tools to assist and improve student learning experiences prior to clinical sessions (24). Feedback from the use of AR and VR tools can be collected and used to refine the models and further enhance learning experiences. Another example of the potential integration of design thinking in dental specialty education can focus on students' lack of competence in diagnosing rare cases in oral radiology and oral medicine. Through the empathise and define phases, educators may notice students' lack of confidence in diagnosing rare diseases since they do not encounter these patients routinely in daily clinical practice. Ideation can then be formulated to create artificial intelligence tools that assist students in interpreting oral radiographs and images, providing a list of possible differential diagnoses for students to further evaluate and make judgments (25, 26). This could be beneficial in helping novices perform tasks with confidence.

As with any new technology, introducing design thinking applications in dentistry poses certain challenges. These challenges encompass “training the trainers” and ensuring educators' proficiency in design thinking methodology, necessitating additional time, resources, and budgets. Moreover, institutional leadership must grasp how to tailor educational methods to learner-centred and patient-centred approaches. Despite these hurdles, mounting evidence underscores the advantages of incorporating design thinking in healthcare education, notably in fostering learners' professional and personal growth, empathy, and creativity.

## Challenges and future direction

Embedding design thinking into educational frameworks is crucial for preparing learners to tackle problems in complex healthcare settings (8). However, integrating design thinking in dental education faces several challenges. First, the faculty members must be equipped with knowledge and processes to utilise the design thinking approach in teaching and learning activities. The faculty development program must be arranged to cultivate the necessary skills and knowledge. Second, to effectively achieve the outcome, the curriculum integration of this teaching method should be well-planned and aligned with program learning outcomes and objectives. Hence, implementing design thinking could add value to learning goals rather than being an extracurricular activity to equip learners with creative and critical thinking skills. Third, integrating design thinking in dental education requires considering resource allocation, including dedicated staff, thematic focus, and physical resources to support these learning activities. Lastly, not only the support to integrating design thinking in the dental curriculum but also the assessment methods for probing the achievement of learning outcomes from those topics/courses used design thinking should also be considered and well-developed to align with the expected skills and competencies.

Although several publications indicate the use of design thinking in a medical education context, employment in dental education could differ in detail. More studies are required to elucidate the effectiveness and appropriateness of integrating design thinking into the dental curriculum to determine its benefits. The example of best practice could set the goals for how to utilise this learning activity in order to achieve the best-expected learning outcome. As in dental education, implementing design thinking must be based on patient-centred design to innovatively address the needs of diverse patients. Taken the aforementioned together, design thinking can be effectively integrated into dental education to foster a new generation of dentists with an innovative mindset for addressing the evolving landscape in dental care and service.

## Conclusion

Design thinking can be effectively integrated into dental education to foster a critical understanding of patients’ needs, which is essential for providing person-centred care. This approach promotes critical and creative thinking skills necessary for navigating the complexities of modern healthcare. By equipping dental learners with design thinking and critical skills, learners are better equipped to find innovative and person-centred solutions for their patients. Additionally, design thinking aligns with cognitivist and constructivist learning principles, emphasising higher-ordered thinking, collaboration, and active engagement, which enhances interdisciplinary teamwork. The iterative nature of design thinking also facilitates continuous feedback and improvement. In summary, integrating design thinking into dental education enriches the learning experience and prepares future professionals to be innovative, empathetic, and adaptable, ultimately improving patient outcomes.
